# The Use of Phosphate of Ammonia in Gout and Rheumatism

**Published:** 1846-02

**Authors:** T. H. Buckler

**Affiliations:** Baltimore


*On the Use of Phosphate of Ammonia in Gout and Rheumatism.* By T. H. Buckler, M. D., of Baltimore. (From the American Journal of Medical Sciences.)

The following extracts from the article of Dr. Buckler will be read with interest, and, if the results should be sustained by the experience of others, may prove of great practical utility. The pathology of the fluids, long neglected, has been lately cultivated with success, and this may be regarded as one of the first attempts to apply the results it has afforded to practice.

"Both diseases are frequently associated with what is called the uric or lithic acid diathesis; that is to say, when a man has a gouty or rheumatic habit, it is generally found that lithic acid is in excess in the secretions of his skin and kidneys. When an individual labours under an acute attack of gout or rheumatism, his recovery is generally heralded by a redundant deposit of lithic acid in his urine. This harbinger of a favorable termination to the disease may happen on the second day of his attack, or on the sixth week, as may be; but whenever it does appear, it may very safely be said that the patient is convalescent.

By what mode this acid is eliminated, or what accident it is which determines its separation, we are unable to say; it stands merely as an isolated fact that by some chemical or vital change taking place, uric acid is separated in great quantity and the individual is relieved. The urine in the course of such an attack may be examined and found as clear as water, and the fluid passed ten or twenty hours after, so loaded with lithic acid as to resemble the washings of a wine cask or beer barrel. From whence is this enormous quantity of lithic acid so suddenly derived? Not from any sudden defect of assimilation occurring in the course of the disease, or from the solids of the body. It is most likely then derived from the blood; but uric acid cannot have existed there in a free state, or it would have been passed from day to day. If then it existed in the blood, it must have been in some state of combination with soda, or lime, or both. And this is the more likely, when we reflect, that the concretions and thickenings which take place in the fibrous, cartilaginous, and white tissues generally, as before stated, are owing to the deposit in them of soda and lime in variable proportions with lithic acid. Taking into account these two prominent facts above stated, namely, the excess of lithic acid found in the urine at the period of convalesence from an attack of acute gout or rheumatism, and the subsequent deposit of soda and lime in the white tissues, it occurred to me, that during the existence of these diseases, the lithic acid might exist in the blood in a state of combination with soda and lime in the form of insoluble compounds, which the kidneys and skin refuse to eliminate. If, then, any agent could be found capable of decomposing the lithates of soda and lime existing in the blood, and of forming in their stead two soluble salts that would be voided by the kidneys and skin, we should thereby get rid of the excess of fibrin in the blood, the symptomatic fever and the gouty and rheumatic inflammation, wherever seated, which have been excited by the presence of these insoluble salts. It occurred to me that *phosphate of ammonia* might be the agent, provided it could be given in doses sufficient to answer the end without producing any unpleasant physiological symptoms. If our theory were true, phosphate of ammonia seemed to be the proper reagent, for it would form in place of the insoluble lithate of soda, two soluble salts, the phosphate of soda, which is remarkably soluble, and the lithate of ammonia, which is also soluble, and both capable of being readily passed by the skin and kidneys. The excess of uric acid would thus be got rid of in the form of lithate of ammonia; and the soda floating in the round of the circulation, (instead of being deposited, as it were, like an alluvial formation in the substance of the fibrous and cartilaginous tissues,) would be taken up by the phosphoric acid and eliminated from the circulation. Based on this theory I determined to try this salt, and it was not long after that a favorable opportunity presented itself.

Case IX.—John Conolly, ætat. 44, entered the hospital June 20th, 1845. He has had more or less rheumatism for the last 12 years. At the time of entrance, he was suffering with an attack in his ankles, knees, and wrists. All these joints were more or less swollen and painful on motion, particularly the wrists, and had been in this condition for six months. The joint of the middle finger of his right hand is anchylosed from a previous attack. He was also suffering from a sclerotitis in both eyes, the left being most diseased; this commenced four weeks previous to his entrance.—There were also traces of an old iritis in his left eye. He was ordered salts and colchicum, blue mass and opium, &c., with various local treatment to his eyes. His rheumatism under this treatment improved a little, but his eyes remained in the same condition. On October 26th, he was ordered phosphate ammoniæ, gr. xij three times daily; and he still continued the salts and colchicum. In three days, his eyes were nearly clear; he had lost all pain, and there was but little injection left. The improvement in his joints was not so perceptible till the end of two weeks. At that time the swelling had much subsided, and he expressed himself as being nearly free from pain. On November 25th, his condition is as follows: he walks well; the swelling in all his joints, except the right knee, has subsided, and he still has some pain on motion, in this joint and the left ankle. He has exposed himself to cold, and has had, once or twice, an increase of inflammation in his eyes, but in two or three days, the injection would subside as before. At present there is no pain, the right eye is clear, but the left has still some injection in the sclerotic coat.

Case X.—Wm. Taylor, ætat. 40, has had rheumatism for the last seven years. Nearly all that time he has been in the hospital in bed, unable to go about the ward without the assistance of crutches. He has taken various remedies, but with slight and temporary benefit. He has suffered more or less in all his joints, but principally his knees and wrists, which have been so much swollen as scarcely to admit of any motion. He has been taking the phosphate of ammonia for the last two months, grs. xij three times daily. He says he is better than he has been for the last five years. The swelling has subsided in a remarkable manner, particularly about his fingers, which are more pliant and useful than they have been since his entrance.

Case XII.—Eliz. J., a woman, ætat. 28, enjoying habitually good health, of a tolerably robust frame, a chamber maid, entered the Baltimore Infirmary on the 3d of November; fourteen days previously she had gone some distance to set up with a sick friend. The evening was inclement, and heated by exercise, she stepped over her ankles into a puddle; her feet were thoroughly wet; perished, as she said; she remained five hours in these wet feet, and felt sick; had headache, nausea, and contusive pains in her back; she returned home, and the next day was scarcely able to go about the house; that night she had a chill, followed by fever and pains in all her articulations. She remained at home in bed without any treatment other than rubbing herself and using mustard plasters, until she came to the hospital.

Dorsal decubitus. Arms and wrists flexed as well as knees; all the articulations of the fingers tense and swollen; wrists doughy and tender; left knee much swollen; complains of both shoulders and left hip; screams when moved in bed; face flushed, expressive of fear and suffering; skin hot, moist; pulse small, tense, 120; dry, hacking cough, which causes her to weep from the motion it produces; slightly prolonged sound with first sound of heart; urine, small in quantity, high-colored and irritating. Venesection ℥xx; cups to præcordial region, No. iv; sulph. magnes. ℥i; vin. colchici ʒij; aquæ ℥viij. A tablespoonful every 4 hours. Phosphat. ammon. grs. x three times a day. Dover's powder grs. xv at night.

I need not detail the changes in this case from day to day. The salts and colchicum were continued for four days, when they produced hyperpurgation and nausea; they were discontinued, and then ten drops of wine of colchicum given twice daily. This also producing nausea, was withdrawn after two days, and the phosphate of ammonia, increased to twenty grs. three times a day, alone persisted in. There was steady improvement from day to day until the 18th. The pulse fell to 72; the rheumatism shifted from joint to joint, but daily its intensity was less and less. The sounds of the heart were normal on the 16th; the urine pale and abundant; the skin moist and without heat; the appetite good; and on the 18th she was, as she said, as "supple as an eel," and proved it by dancing. On the night of the 20th, she was up while in a state of perspiration with a sick woman, and had a slight relapse; the finger joints again became puffy and swollen and stiff. But confinement to bed and the phosphate sufficed in two days to relieve this. I called to see her on the 24th; she is well with her friends; complains of no pain; looks a little pale; the heart is normal, and the joints natural in size.

